# Pernicious Anæmia and Its Diagnosis, Especially in Obscure Nervous Conditions

**Published:** 1907-02-02

**Authors:** 


### Points in Diagnosis.

### PERNICIOUS ANÆMIA AND ITS DIAGNOSIS, ESPECIALLY IN OBSCURE NERVOUS CONDITIONS.

The epithet " pernicious " is perhaps unfortunate in connection with this disease, because to many it conveys the idea of " hopeless," or even of " rapidly fatal." This is erroneous, for many patients suffering from pernicious anæmia survive for years, and sometimes the improvement under treatment is remarkable, even though such improvement be maintained only for a time. The condition is, of course, less hopeful the later it is diagnosed, and the later suitable treatment is adopted. It behoves us all to be able to diagnose the condition early, and to diagnose it we must be on the look-out for it.

The clinical picture in a typical case is well known; the pale yellow waxy appearance of the face and body, the lassitude and weakness, and particularly the absence of wasting, or rather the persistence of a flabby stoutness, are the three most obvious signs of it. It is, however, late in the disease before these three signs are all well marked; in the earlier stages the condition may not even be suspected unless a blood examination is made to determine the nature of an anæmia which may not as yet be very marked. By so doing it will be found that pernicious anæmia is by no means excessively rare. It is true that many suspicious cases will prove negative, and patients may be put to the expense of blood examination apparently without cause; but in the negative cases it is well worth while to know that there is no incipient pernicious anæmia, and in the positive cases the early diagnosis is all-important. It is so easy nowadays to have a complete blood examination made at a small cost by sending specimens to one of the clinical research laboratories who supply all the apparatus needed for the purpose.

The pathognomonic features of the blood are, first and foremost, the high colour index of the anæmic blood; and, secondly, the appearance of the blood films. The " colour index " is the ratio of the hæmoglobin to the red corpuscles. In healthy blood the amount of hæmoglobin is arbitrarily called 100, the normal number of red corpuscles being also called 100; thus in healthy blood the colour index is $\frac{100}{100}$, or 1. In almost every form of anæmia, except pernicious, the hæmoglobin is diminished either in the same proportion as are the red corpuscles, or else in greater degree, so that the colour index is either 1 or less than 1. In pernicious anæmia, on the contrary, the hæmoglobin, though diminished, is less diminished than are the red corpuscles, so that the colour index is greater than 1. For example, if the red corpuscles were down to 30 per cent. of normal, the hæmoglobin might only be down to 40 per cent. of normal, so that the colour index would be $\frac{40}{30}$ or 1.3. This high colour index in the presence of anæmia is pathognomonic of pernicious anæmia.

If circumstances prevent us from estimating the hæmoglobin and the number of red corpuscles per cubic millimetre, it is still possible to make a shrewd guess at pernicious anæmia if we make films of the blood and examine them under a high power. Not only will many poikilocytes be seen, but there will be great variations in the sizes of the corpuscles, with a predonderance of large red blood discs or megalocytes. An anæmia with preponderance of megalocytes in the blood films is almost certainly pernicious anæmia; but this method of diagnosis is less reliable than is that of estimating the colour index.

When pernicious anæmia is only suspected in the late, and therefore more hopeless, cases, its diagnosis will be from other forms of severe anæmia, such as cancerous cachexia, particularly cancer of the stomach; syphilitic cachexia, malarial cachexia, lardaceous disease, fungating endocarditis, chronic tubal nephritis; the anæmia produced by intestinal parasites such as bothriocephalus latus or ankylostoma duodenale; myxœdema, rectal polypi, menorrhagia, Addison's disease. It is not with these that we will deal just now, but rather with the difficulties in the earlier stages; and of the earlier conditions in which other symptoms attract the attention so much that pernicious anæmia may never occur to one, those of nervous diseases are very important.

It is no new observation that various slowly progressive degenerations can occur in the spinal cord in cases of pernicious anæmia. It is not generally recognised, however, that such degenerative cord changes are by no means restricted to the late stages, but may occur so early as to seem to be the primary condition. The spinal changes may be so gradual that at first neurosis may be diagnosed. In obscure cases of cord disease associated with even only slight anæmia it is well to examine the blood. Dr. G. Lovell Gowland has recently published a number of such cases. The following is another instance.

An elderly lady began to feel weak in her legs, and suffered from severe but obscure pains in different parts of her back, abdomen, and lower limbs. She felt " as if she were about to become paralysed." She went to two different special hospitals for nerve diseases, and saw the best nerve specialists upon many occasions. After careful examination none of the distinctive signs of an organic nerve lesion could be found. She was assured that the trouble was functional and not organic, and she felt reassured for a time. For over two years, however, the pains not only persisted, but got gradually worse, until finally she was unable to endure the fatigue of dragging herself as far as the hospital. It was only at this time that her plantar reflex, previously flexor, became at first doubtfully, and then definitely, extensor (Babinsky's sign). The presence of organic disease of the cord was further confirmed by the development presently of retention of urine with overflow, necessitating catheterisation twice daily. At this time the blood was examined; the hæmoglobin was 58 per cent. of normal, the red corpuscles were 32 per cent., so that the colour index was $\frac{58}{32}$, or 1.8. The films were typical, and there was no leucocytosis. The condition was pernicious anæmia; and it was then recalled that her general appearance had been compatible with the presence of pernicious anæmia for over two years past.

This case by itself would be of little value; but Dr. Gulland says he has found many similar cases, in which pernicious anæmia was lost sight of on account of the prominence of nerve symptoms. Remembering this, many of us are sure to come across similar instances in practice; but the diagnoses will not be made early unless blood-counts are carried out in a considerable number of cases, in which the anæmia falls short of the lemon-yellow colour of typical pernicious anæmia.

